# Cell-associated hemolysis activity in the clinical strain of *Pseudomonas fluorescens MFN1032*

**DOI:** 10.1186/1471-2180-10-124

**Published:** 2010-04-24

**Authors:** Daniel Sperandio, Gaelle Rossignol, Josette Guerillon, Nathalie Connil, Nicole Orange, Marc GJ Feuilloley, Annabelle Merieau

**Affiliations:** 1 Laboratory of cold microbiology signals and the microenvironment, LMDF-SME, UPRES EA 4312, University of Rouen, 55 rue Saint Germain, 27000 Evreux, France

## Abstract

**Background:**

MFN1032 is a clinical *Pseudomonas fluorescens *strain able to grow at 37°C. MFN1032 cells induce necrosis and apoptosis in rat glial cells at this temperature. This strain displays secretion-mediated hemolytic activity involving phospholipase C and cyclolipopeptides. Under laboratory conditions, this activity is not expressed at 37°C. This activity is tightly regulated and is subject to phase variation.

**Results:**

We found that MFN1032 displays a cell-associated hemolytic activity distinct from the secreted hemolytic activity. Cell-associated hemolysis was expressed at 37°C and was only detected *in vitro *in mid log growth phase in the presence of erythrocytes. We studied the regulation of this activity in the wild-type strain and in a mutant defective in the Gac two-component pathway. GacS/GacA is a negative regulator of this activity. In contrast to the *Pseudomonas fluorescens *strains PfO-1 and Pf5, whose genomes have been sequenced, the MFN1032 strain has the type III secretion-like genes *hrc*RST belonging to the *hrpU *operon. We showed that disruption of this operon abolished cell-associated hemolytic activity. This activity was not detected in *P.fluorescens *strains carrying similar *hrc *genes, as for the *P. fluorescens *psychrotrophic strain MF37.

**Conclusions:**

To our knowledge this the first demonstration of cell-associated hemolytic activity of a clinical strain of *Pseudomonas fluorescens*. Moreover, this activity seems to be related to a functional *hrpU *operon and is independent of biosurfactant production. Precise link between a functional *hrpU *operon and cell-associated hemolytic activity remains to be elucidated.

## Background

*Pseudomonas fluorescens *is a highly heterogeneous species, as shown the extensive literature on the taxonomy and phylogeny of this species [1-4]. These studies include saprophytic, rhizopheric and phytopathogenic strains of *P. fluorescens*, illustrating the ubiquity of this species. Most studies describe *P. fluorescens *as a psychrotrophic bacterium unable to grow at temperatures greater than 32°C and therefore as an avirulent bacterium in humans. Nevertheless, previous studies of the infectious potential of *P. fluorescens *have demonstrated that the rifampicin spontaneous mutant MF37 [5] derived from the environmental psychrotrophic strain MF0 [6] can bind specifically to the surface of neurons and glial cells [7]. This adhesion to the host cell is associated with the induction of apoptosis and necrosis in glial cells [8]. Lipopolysaccharides (LPS) produced or released by *P. fluorescens *have a clear role in cytotoxicity, but other factors released at the same time during adhesion also seem to be essential for the virulence of this bacterium [9]. Thus the various enzymes secreted by this species may also be considered as potential high virulence factors [5].

We recently demonstrated that the clinical strain MFN1032 is a *Pseudomonas fluorescens sensus stricto *Biovar1 strain able to grow at 37°C [10]. This strain has hemolytic activity mediated by secreted factors, similar to the hemolytic activity seen for the opportunistic pathogen *Pseudomonas aeruginosa*, involving phospholipase C (PlcC) and biosurfactant [11]. Under specific conditions, MFN1032 forms colonies of phenotypic variants, which are defective in secreted hemolysis. Spontaneous mutations of the genes encoding the two-component regulatory system GacS/GacA have been identified as the cause of phenotypic variation in one such group of variants. We hypothesized that phenotypic variation increases the virulence potential of this strain. However these group variants (group 1 variants) do not produce secondary metabolites and have impaired biofilm formation [12]. Then, these results suggested that virulence of MFN1032 is not dependent solely on secreted factors or LPS and thus must involve other factors.

Some bacterial virulence factors are only expressed in the presence of eukaryotic cells. This is the case of the type III secretion system (TTSS), one of the most frequently described contact dependent secretion systems in *Pseudomonas*. TTSSs are found in many Gram-negative pathogens. They allow the direct translocation of bacterial effector proteins into the cytoplasm of eukaryotic host cells. *P. aeruginosa *uses the TTSS to translocate four effector proteins (ExoS, ExoT, ExoU, and ExoY) with antihost properties [13]. The *P. aeruginosa *TTSS consists of nearly 40 genes, regulated in a coordinated manner and encoding structural components of the secretion and translocation machinery, effectors proteins, and regulatory factors [14]. Transcription of the TTSS is induced under calcium-limited growth conditions or following intimate contact of *P. aeruginosa *with eukaryotic host cells [15]. *Pseudomonas syringae *pv. *tomato *DC3000 is a phytopathogenic bacterium that harbors a gene cluster *hrp *(for hypersensitive reaction and pathogenicity). This *hrp *cluster is essential for pathogenicity in susceptible plants and for the ability to elicit the hypersensitive reaction in nonhosts or resistant cultivars of host plants [16]. *hrp *genes are expressed *in planta *or *in media *mimicking plant apoptotic conditions [17]. Sequence analyses have uncovered a shared subset of nine *hrp *genes that were renamed *hrc *(for *hrp *and conserved) and that encode proteins homologous to *Yersinia ysc *gene products [18]. The existence of these genes suggests evolutionary conservation of molecular mechanisms of pathogenicity used by both mammalian and phytopathogenic bacteria [19].

In *P. fluorescens*, the presence of the *hrc *genes belonging to *hrpU *operon depends on the strain. The feature of TTSS and the origin of *hrc *genes remain to clarify in this species [20-23].

In the present study, we describe the detection of cell-associated hemolytic activity of P. fluorescens MFN1032 in contact with sheep erythrocytes. This hemolytic activity was compared with the hemolytic activity of other *P. fluorescens *strains: a spontaneous MFN1032 *gac*A mutant and the opportunistic pathogen *Pseudomonas aeruginosa *CHA [24]. Cell-associated hemolytic activity and its regulation were compared with the activity and regulation of the previously described secreted hemolytic activity of MFN1032. We then looked for *hrc *genes in our strain and determined their role in the cell-associated hemolytic activity of MFN1032, using *hrpU *operon disruption mutant.

## Results

### MFN1032 displays cell-associated hemolytic activity

Hemolytic activity of *Pseudomonas fluorescens *biovar I MFN1032 and *Pseudomonas aeruginosa *CHA (positive control for TTSS-mediated hemolysis) was measured by the technique employed by Dacheux [25], adapted as described in methods.

**Figure 1:**
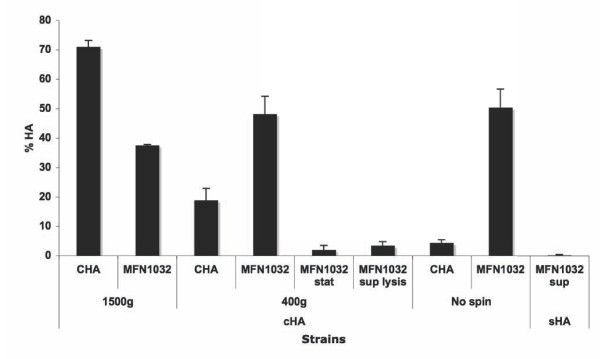
**Comparison of the hemolytic activity of MFN1032 and CHA and characterization of MFN1032 cell-associated hemolysis**. Hemolysis of RBCs (% HA) incubated with MFN1032 and CHA, at 37°C and with a multiplicity of infection (MOI) of 1. Cells were subjected or not to centrifugation at 1500 g or 400 g for 10 min to enhance cell-cell contact. cHA indicates cell-associated hemolytic activity and sHA indicates secreted hemolytic activity. MFN1032 sup indicates MFN1032 cell-free supernatant. MFN1032 stat indicates MFN1032 cells in stationary growth phase. MFN1032 sup lysis indicates supernatants obtained after RBC lysis by MFN1032. Hemolytic activity was measured as described in the materials and methods. Results are means of at least three independent experiments. Standard deviation is shown.

Bacteria were grown at 37°C to mid exponential growth phase and were used at a multiplicity of infection (MOI) of 1, without spin (which enhance contact between bacteria and RBCs). CHA induced lysis of 5% of red blood cells (RBCs) and MFN1032, 50% lysis, within 1 hour at 37°C. Hemolytic activity of CHA was increased by a 10 min centrifugation at 400 g (20% lysis) or 1500 g (70% lysis). By contrast, the hemolytic activity of MFN1032 was unchanged after a 10 min centrifugation at 400 g and reduced by centrifugation at 1500 g (35% lysis) (Figure 1). For further experiments we used a 10 min centrifugation at 400 g since this protocol is allowing close contact between bacterial cells and RBCs and appears compatible with maximum lysis by MFN1032. Supernatants from MFN1032 cells tested in the same conditions had no hemolytic activity. Additionally, we collected supernatants from RBC lysed by MFN1032. Supernatants were filtered and incubated with fresh RBCs for 1 h at 37°C. This supernatant from lysed RBC samples did not induce further RBC lysis. Thus, the factor mediating RBC lysis is not a factor released into the supernatant, but is dependent on the presence of MFN1032 cells.

**Figure 2:**
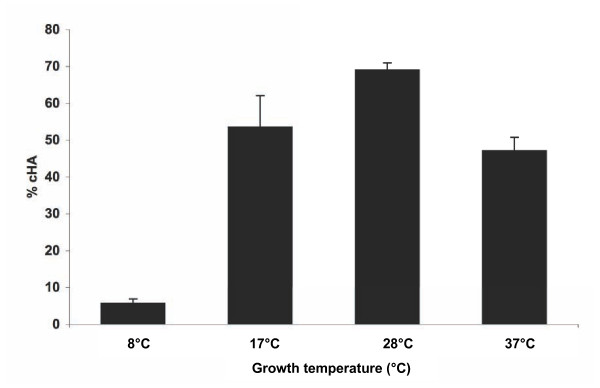
**Influence of growth temperature on MFN1032 cell-associated hemolytic activity**. Cell-associated hemolytic activity (cHA %) was measured for MFN1032 grown at 8°C, 17°C, 28°C (optimum growth temperature) or 37°C, as described in the materials and methods. Results are means of at least three independent experiments. Standard deviation is shown. Contact was enhanced by centrifugation at 400 g for 10 min.

MFN1032 cells from cultures grown to the exponential growth phase at various temperatures were incubated with RBCs for 1 h at 37°C. MFN1032 bacteria grown at 17°C and 37°C showed the same levels of hemolysis (50% of RBCs lysed), whereas bacteria grown at 8°C were almost devoid of hemolytic activity (5% lysis). The maximal hemolytic activity of MFN1032 was observed at 28°C (70% lysis), the optimal growth temperature of this strain (Figure 2).

### Lysis of RBCs is caused by a pore-forming toxin from MFN1032

**Figure 3:**
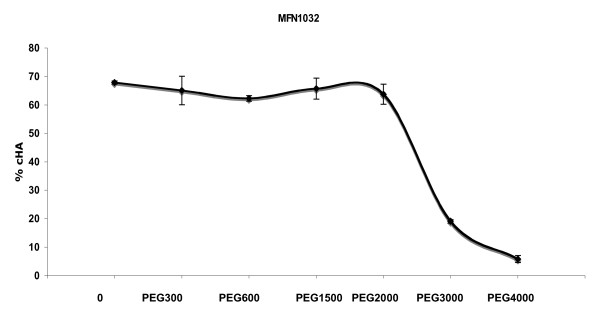
**Protection of RBCs from cell-associated hemolysis by osmoprotectants**. Omoprotectants were added at a final concentration of 30 mM. All experiments were performed at least three times in triplicate. MFN1032 was grown at 28°C. Standard deviation is shown.

We investigated the nature of the factor involved in RBC lysis by osmoprotection experiments. Osmoprotectants protect RBCs against osmotic shock provoked by bacterial pore-forming toxins. We used different sized molecules in hemolysis experiments to estimate the size of the pore formed in the RBC membrane (Figure 3). We did not observe any effects on hemolysis with PEG300, PEG600, PEG1500 or PEG2000. Molecules larger than PEG2000 protected against MFN1032 cell-associated hemolysis as observed for PEG3000. A maximal level of protection was reached with PEG4000, resulting in the protection of 90% of RBCs against this hemolytic process. Based on these results, we estimated the size of the pore formed in RBC membranes by MFN1032 is between 2.4 nm and 3.2 nm.

### MFN1032 cell-associated hemolytic activity is higher than in other *Pseudomonas fluorescens *strains

We tested the hemolytic activity of MFN1032, MFY162 (a clinical isolate of *Pseudomonas fluorescens *Biovar I), MFY161 and MFY163 (clinical isolates of *Pseudomonas mosselli) *[10], MF37 (*Pseudomonas fluorescens *biovar V, derived from a psychrotrophic environmental strain) and C7R12 (a psychrotrophic rhizospheric strain) cells at 28 and 37°C.

**Figure 4:**
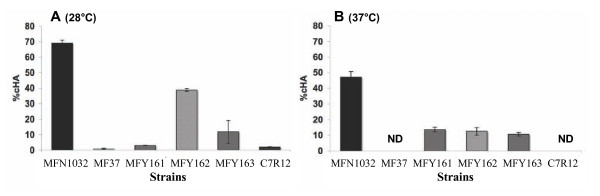
**Cell-associated hemolytic activity of different fluorescent *Pseudomonas *strains**. Cell-associated hemolytic activity (cHA %) was measured as described in the materials and methods. Results are means of at least three independent experiments. Standard deviation is shown. **A**: Hemolysis of RBCs incubated with MFN1032, MF37, C7R12, MFY161, MFY162, MFY163 at 28°C and MOI of 1. Contact was enhanced by centrifugation at 400 g for 10 min. **B**: Hemolysis of RBCs incubated with MFN1032, MF37, C7R12, MFY161, MFY162, MFY163 at 37°C and MOI of 1. Contact was enhanced by centrifugation at 400 g for 10 min. ND: not determined. MF37 and C7R12 were unable to grow at 37°C.

The hemolytic effect of MFN1032 cells was much higher than the other strains tested, at both growth temperatures (Figure 4). At 28°C, MFY162 was the only other strain showing high levels of hemolytic activity (40% lysis); MFY161 and MFY163 displayed only weak hemolytic activity (5-10% lysis). All clinical isolates showed some hemolytic activity (15% lysis) at 37°C, but at a lower level than that observed for MFN1032 one's. The environmental strains tested were not hemolytic at 28°C and did not grow at 37°C.

The hemolytic activities of MFN1032, MFY162 and MFY161, were maximal at their optimal growth temperature (28°C for MFN1032 and MFY162, 37°C for MFY 161). The hemolytic activity of the strain MFY163 was the same at 28°C and 37°C.

### Involvement of the Gac two-component system on cell-associated hemolytic activity

**Figure 5:**
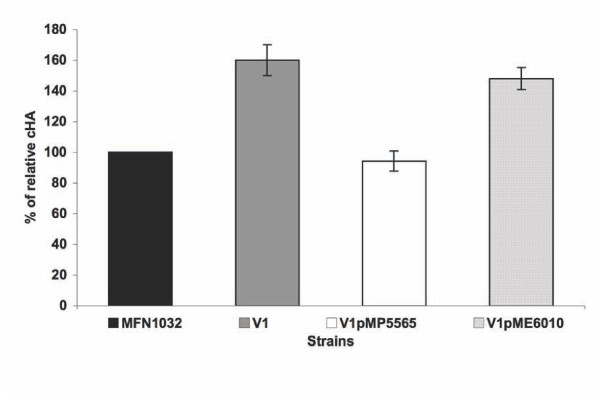
**Effect of GacA on MFN1032 cell-associated hemolytic activity**. Cell-associated hemolytic activity (cHA) for MFN1032 cells, V1 (*gac*A mutant) and V1 cells carrying the *gacA *gene-containing plasmid (pMP5565), or the parental plasmid pME6010 used as a control. The cHA of MFN1032 was taken as the reference value (100%); results are expressed as percent of this value (% relative cHA). The strains were grown at 28°C. Results are means of at least three independent experiments. Standard deviation is shown. Contact was enhanced by centrifugation at 400 g for 10 min.

We investigated the possible involvement of the Gac two-component system in the regulation of this cell-associated hemolytic activity using a group1 variant of MFN1032, V1. This variant strain is a *gacA *mutant and has impaired secreted hemolytic activity [12]. V1 was tested with or without transformation by electroporation with plasmid carrying the *gacA *gene (pMP5565) or the parental plasmid pME6010, as a control [26] (Figure 5).

The non-transformed V1 strain displayed enhanced hemolytic activity (160% lysis), using MFN1032 as a reference value (100%). Introduction of a *gacA *gene in V1 cells by electroporation with pMP5565 restored wild-type hemolytic levels.

### MFN1032 has *hrc*RST genes

The lysis of RBCs by a number of bacteria requires the formation of a pore in the erythrocyte membrane by TTSS protein. To determine whether TTSS-like genes are present in MFN1032 and MF37, we used PCR primers targeting *hrpU *operon, (so called U operon of the *hrp *cluster of type I) encoding the conserved core proteins of fluorescent *Pseudomonas *TTSS, described by Mazurier *et al*. [23]. The region amplified by these primers includes the 3'end of *hrc*R, *hrc*S and the 5'end of *hrc*T.

A single fragment of 0.9 kb was obtained for MFN1032 and MF37 and cloned with the pMOS kit. Fragments were sequenced by Genome Express (France). Sequences were registered in the Genbank database (accession number: EU811174 for MFN1032 and FJ694188 for MF37) and named "*hrc *operon", partial sequence. These sequences predict an 87 residues HrcS protein in these two strains.

A NCBI nucleotide and protein database search showed that the putative HrcS from MFN1032 was very similar to the putative MF37 HrcS (90% identity) and to RscS (94% identity), a type III secretion protein from the *Pseudomonas fluorescens *strain SBW25 (belonging to the HrcS/YscS/FliQ family), but is more distantly related to the HrcS of C7R12 (73.9% identity) (Table 1). The *P. aeruginosa *PAO1 FliP partial protein showed 47% identity.

**Table 1:** Comparison of the MFN1032 Hrc*S *sequence with other Hrc-like sequences

Strain	HrcS-like GenBank number	% Identity to HrcS MFN1032
*P.fluorescens *MFN1032	Putative HrcS ACE88958	-
*P.fluorescens *SBW25	Putative type III secretion membrane protein RscS CAY46985	94%
*P. fluorescens *Pf-5	Flagellar biosynthesis protein FliQ AAY90949	NS
*P. fluorescens MF37*	Putative HrcS ACO58571	90%
*P. fluorescens *Pf0-1	Flagellar biosynthesis protein FliQ ABA73293	NS
*P. fluorescens C7R12*	Putative HrcS CAC24707	74%
*P.aeruginosa PAO1*	FliP AAG04835	47%
*Pseudomonas syringae *pv. *tomato *str. DC3000	Type III secretion protein HrcS AAO54916	76%
*Pseudomonas syringae *pv. *phaseolicola *1448A	Type III secretion component protein HrcS CAE53643	74%

NS: not significant (< at 40%)

### Effect of disruption of the *hrpU *operon

**Figure 6:**
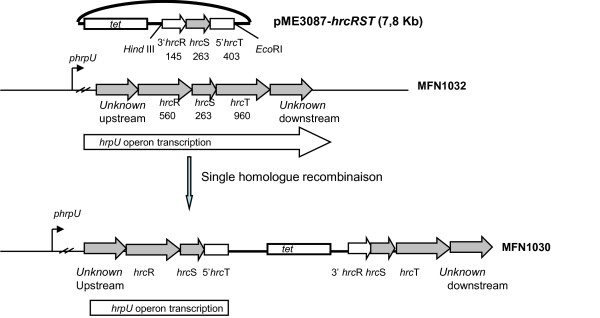
**Construction of MFN1030 *hrpU *operon disrupted mutant**. *phrp*U indicates the promoter of *hrpU *operon. *tet *is the tetracycline resistance gene of pME3087. The length of each *hrc *gene is indicated below its designation (in base pairs). 3'*hrc*R indicates *hrc*R gene deleted in 5'-end and 5'*hrc*T indicates *hrc*T gene deleted in 3'-end. The arrow above the genes represents the operon transcription. A bold line represents DNA from pME3087. *Hind*III and *Eco*RI are enzymes used to clone *hrc*RST in pME3087. *Unknown *indicates putatives *hrc *genes located upstream or downstream *hrc*RST genes.

**Figure 7:**
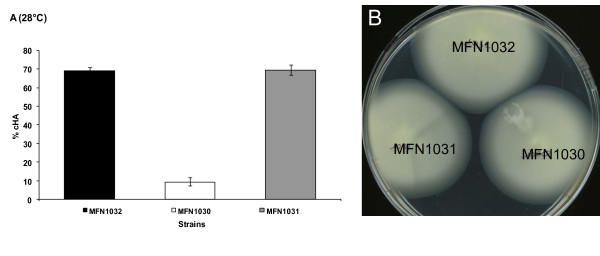
**Cell-associated hemolytic activity and swimming motility of MFN1032, MFN1030 (MFN1032 *hrc*RST-disrupted mutant) and MFN1031 (revertant)**. **A**: Hemolysis of RBCs incubated with MFN1032, MFN1030 and MFN1031 at 28°C and a MOI of 1. Results are means of at least three independent experiments. Standard deviation is shown. Contact was enhanced by centrifugation at 400 g for 10 min. **B**: Swimming motility of MFN1032, MFN1030 and MFN1031. Swimming motility was determined, as described in the methods, on 0.3% LB agar after 16 h of incubation at 28°C. MFN1032, MFN1031 and MFN1030 formed concentric halos corresponding to swimming motility.

We investigated a possible link between this *hrpU *operon and the cell-associated hemolytic activity of MFN1032. We used a mutant strain, MFN1030, in which the *hrpU *operon was disrupted, to determine whether TTSS proteins are required for the hemolytic activity observed in MFN1032. In this construction, the single homologue recombination provokes at least the deletion of the 5'-end of *hrc*T (58% of HrcT) and of genes situated downstream *hrc*T in this operon (Figure 6). We observed an almost total loss of cell-associated hemolytic activity (10% lysis) in the mutant strain. Revertant of MFN1030, the strain MFN1031, had a restored hemolytic phenotype, showing activity levels similar to those of MFN1032 (Figure 7A). These results demonstrate a link between the *hrpU *operon and this cell-associated hemolytic activity in *P. fluorescens *MFN1032.

Given the homology between *hrcS *and *fliP*, we investigated the potential role of *hrc*RST genes in flagellar synthesis. The effect of disrupting the *hrpU *operon in MFN1030 was measured in swimming mobility assays, as described in the methods. At 28°C, we observed no differences in swimming ability between MFN1032, MFN1030 and MFN1031 (Figure 7B), suggesting that disruption of this operon has no effect on flagella motility.

## Discussion

To our knowledge this is the first study to demonstrate cell-associated hemolytic activity in clinical isolates of a *Pseudomonas fluorescens. P.fluorescens *MFN1032 cell in exponential growth phase displayed hemolytic activity at 37°C, whereas no hemolytic activity was detected using MFN1032 supernatant. This hemolytic activity was thus dependent on the presence of MFN1032 cells. MFN1032 cells caused hemolysis of RBCs without requiring prior centrifugation to reduce the distance between bacterial and red cell membrane below a critical threshold. Such a centrifugation step has previously been shown to be necessary to induce the "contact-dependent" hemolytic activity displayed by several other bacteria, for example *Yersinia *[27], *Shigella *[28] and *Pseudomonas aeruginosa *[25]. In contrast, "induced" hemolysis does not require close RBC-bacterial contact for enteropathogenic *Escherichia coli *EPEC, due to the long EspA (TTSS secreted protein) filaments that form a connection between bacteria and host cells for protein translocation [29,30]. MFN1032 hemolytic activity was not strictly contact-dependent but depended on the presence of MFN1032 cells. We therefore propose the term "cell-associated" hemolytic activity.

This activity is independent of the secreted hemolytic activity previously described for this strain. For all tested conditions, we have previously demonstrated that secreted hemolytic activity only occurs at the end of the exponential growth phase [11]. MFN1032 supernatants were not hemolytic at 37°C *in vitro *and hemolysis was maximal at 17°C. Cell-associated hemolysis measured here was maximal during the exponential growth phase and retrieved at 37°C. Moreover, a *gacA *mutant of MFN1032 (V1), for which several extracellular activities are impaired (including secreted hemolytic activity), showed increased cell-associated hemolytic activity. In psychrotrophic bacteria, most secreted enzymes are generally found at 17°C (critical temperature), whereas membrane-associated activities are enhanced with decreased generation time [6,31]. Thus, the maximum expression of this new hemolytic activity at 28°C (optimal growth temperature) is consistent with a cell surface associated process.

This hemolytic activity is not common to all *Pseudomonas fluorescens *species. Indeed, we only observed significant cell-associated hemolysis in the clinical strains MFN1032 and MFY162 and not in the environmental strains tested. Although our panel of studied strains is limited and can not be considered as representative, the presence of this activity seems to be dependent on strain origin, *i.e *clinical source. Cell-associated hemolytic activity has been rarely observed in environmental strains. Nevertheless, two hemolytic strains showing such phenotype have been described for *Plesiomonas shigelloides *(former *Pseudomonas*) [32].

We amplified TTSS-like genes *hrc*RST from MFN1032 and MF37 cells while *P.fluorescens *PfO-1 and Pf5 strains [21,33] lack the TTSS genes found in related pathogens or plant-associated bacteria. *hrpU *operon-like has previously been found in the *P. fluorescens *strains KD (phytoprotection strain) and SBW25 (biocontrol strain) [22,34]. In one study of a group of fluorescent *Pseudomonas*, TTSS-like genes were detected in 75% of the phytopathogenic but only in 32% of the saprophytic strains tested [23]. The presence of *hrc*RST genes is not systematically correlated to hemolytic activity. Indeed, *P. fluorescens *MF37 and C7R12 have similar *hrc*RST genes to MFN1032 but are not hemolytic. Thus, the presence of these genes does not strictly imply hemolytic function. Lysis is dependent upon the ability of TTSS translocator proteins to form a pore in the erythrocyte membrane causing hemoglobin leakage. The presence of these *hrc*RST genes does not necessarily result in the assembly of a functional TTSS. Some TTSS genes are absent from SBW25 and TTSS virulence genes in KD have been suggested to have been recently acquired horizontally from phytopathogenic bacteria and recycled for biocontrol function [22].

TTSS-dependent lysis of erythrocytes has been observed in a number of bacteria. Contact-dependent hemolysis assays have been used to identify the genes required for a functional *Salmonella *TTSS 1 [35]. MFN1032 cell-associated hemolytic activity shares common features with TTSS-mediated hemolysis. The various mechanisms involved include formation of a pore with an estimated size of 2.4 to 3.2 nm, consistent with TTSS-hemolysis mechanism previously described by Dacheux for *Pseudomonas aeruginosa *[25]. MFN1032 cells did not show this cell-associated hemolysis during the stationary growth phase. Previous studies have shown a negative effect of high cell density, through a RpoS-mediated mechanisms [36] or by quorum-sensing [37], on TTSS gene expression in *Pseudomonas aeruginosa*. We found increased hemolytic activity in the MFN1032 *gacA *mutant (V1). This result suggests that the Gac two-component system is a negative regulator of cell-associated hemolytic activity. Studies on TTSS regulation in *Pseudomonas aeruginosa *have demonstrated that the GacA response regulator inhibits TTSS function and that, in a *gacA *mutant, the TTSS effector ExoS is hypersecreted [38]. Opposite, in *Pseudomonas syringae*, GacA is a positive regulator of the TTSS [39]. The homology between MFN1032 genes and plant-associated TTSS genes is not in favour of a direct negative transcriptional regulation by the system Gac.

To investigate the potential role of TTSS in this hemolytic process, we constructed a mutant with *hrpU *operon disruption, MFN1030, in which hemolytic activity was severely impaired. Hemolysis was restored in revertant MFN1031 cells, with hemolytic activity levels similar to wild type. Thus, cell-associated hemolytic activity seems to require an intact *hrpU *operon. In contrast, *hrpU *operon disruption did not affect swimming motility, suggesting that *hrpU *operon is not involved in flagella biosynthesis. In MFN1030 the single homologue recombinaison event with PME3087-*hrc*RST would result in, at least, a lack of HrcT protein. In *Pseudomonas cichorri*, an insertion of transposon in *hrc*T was described as sufficient to lost virulence on eggplant [40]. This large insertion in MFN1030 would have a polar effect on genes situated downstream this operon. In *Pseudomonas fluorescens*, *hrcRST *genes are highly conserved. Other genes of the *hrpU *operon, however, seem to vary considerably [22,34]. PCR experiments based on SBW25 and KD sequences did not lead to an amplification of any *hrc *genes located downstream or upstream *hrc*RST (data not shown). An experiment of chromosome walking should allow us to identify these genes.

The *hrcRST *genes from *Pseudomonas fluorescens *MFN1032 show a high level of homology with *hrcRST *genes from *Pseudomonas syringae*, a plant pathogen. TTSS-dependent pore formation is due to the insertion of the translocation pores into host cell membranes. In *Pseudomonas syringae*, Hrpz_*psph *_forms pores *in vitro *and is exported by the TTSS. However, when introduced into *Yersinia enterocolitica *cells, this protein is exported via the *Yersinia *SSTT but cannot replace YopB functions and do not cause RBC hemolysis [19]. HrpZ is unable to induce pore formation. Moreover, in the two strains of *Pseudomonas fluorescens *already described no *hrp*Z homologue was found. We tried to amplify this gene with primers design from *hpr*Z from other *pseudomonad*, but without success.

The nature of the TTSS component implicates in the cell hemolytic activity of MFN1032 remains to be determined. We will address this issue in future studies.

## Conclusion

*Pseudomonas fluorescens *MFN1032 is a clinical strain isolate that displays two distinct types of hemolytic activity, described here for the first time. The first type is observed in the cell-free supernatant of rich media cultures at 28°C, whereas the second, cell-associated type of hemolysis, is detected at 37°C in the presence of erythrocytes. This strain has *hrc*RST genes, a feature that is not shared by all *Pseudomonas fluorescens *strains. Our study establishes an unexpected link between these *hrc *genes and cell-associated hemolytic activity. These initial findings are consistent, although not sufficient, to demonstrate that this cell-associated hemolysis is due to a functional TTSS. Investigation of type III effector genes in the genome of this strain and the construction of targeted mutants are now needed to confirm these findings. Nevertheless, this study suggests that certain strains of the highly heterogeneous species *Pseudomonas fluorescens*, which is usually considered to be a saprophytic species, express virulence with characteristic of pathogenic species belonging to the *Pseudomonas *genus. Nevertheless the principal role of this TTSS homologue to the one of plant-associated bacteria is probably not the pathogenicity against endotherms. The first target of this system would rather be unicellular eukaryotes of the rhizosphere, as mycetes or amoebas.

## Methods

### Bacterial strains and culture conditions

The MFN1032 strain was collected from a hospital patient suffering from pulmonary tract infection (expectoration) and was considered to be the cause of the infection. MFN1032 was identified as a *Pseudomonas fluorescens *biovar I strain [10] and was able to grow at 37°C.

CHA is a bronchopulmonary isolate of *Pseudomonas aeruginosa *from a cystic fibrosis patient [24]. This strain induces TTSS-dependent but ExoU-independent oncosis of neutrophils and macrophages. CHA-induced macrophage death results from a pore forming activity that is dependent on the TTSS. Contact dependent hemolysis provoked by CHA requires the same pore forming activity. CHA has a well inducible and tightly regulated TTSS [41], and is used in our study as a positive control of RBC-TTSS hemolysis.

MF37 is a spontaneous rifampicin-resistant mutant of the MFO strain, a psychrotrophic strain of *Pseudomonas fluorescens *biovar V, isolated from raw milk and extensively studied in our laboratory [5].

MFY162 is a clinical isolate of *Pseudomonas fluorescens *Biovar I, MFY161 and MFY163 are clinical isolates of *Pseudomonas mosselli *[10] and C7R12 a *Pseudomonas fluorescens *psychrotrophic rhizospheric strain [42].

These bacteria were cultured in Luria Bertani medium (LB), at various temperatures between 8 and 37°C, with shaking at 180 rpm. When necessary, 20 μg/mL tetracycline or 100 μg/mL ampicillin was added. Bacterial density was determined by measuring optical density at 580 nm (Spectronic Unicam spectrophotometer).

### Swimming motility

Each strain was incubated on LB agar plates for 24 h at 28°C. Plates of LB medium solidified with 0.3% agar were inoculated by stabbing colonies with a toothpick and inserting the end of the toothpick just below the surface of the agar. Three colonies were picked from three plates and incubated at 28°C until a migration halo appeared.

### Hemolysis assay

Hemolysis was performed essentially as described by Dacheux [25]. Sheep red blood cells (RBCs), obtained from Eurobio (France), were washed three times in PBS (pH 7.2, 0.8% NaCl, 0.02% KCl, 0.17% Na_2_HPO_4_, 0.8% KH_2_PO_4_) and resuspended in RPMI-1640 medium without pH indicator (Sigma) at a density of 5 × 10^8 ^RBCs mL^-1 ^at 4°C. Bacteria were grown in LB to an OD_580 nm _of 0.7 - 1.5, centrifuged and resuspended in RPMI-1640 at 5 × 10^8 ^bacteria mL^-1^. Hemolysis assays were started by mixing 100 μL of RBCs and 100 μL of bacteria, which were than centrifuged at 1500 g or 400 g for 10 minutes and incubated at 37°C for 1 h. The release of hemoglobin was measured at 540 nm, after centrifugation, in 100 μL of cell supernatant.

The percentage (%) of total lysis was calculated as follows: % = [(X -B)/(T-B)] × 100, where B (baseline), a negative control, was corresponding to RBCs incubated with 100 μL of RPMI-1640, and T, a positive control, was corresponding to total RBCs lysis, obtained by incubating cells with 0.1% SDS. X is the OD value of the analysed sample.

When indicated, RBCs were resuspended in 60 mM sterile solutions of osmoprotectant in RPMI-1640, to give a final concentration of 30 mM. For these experiments, a control of hemoglobin precipitation in presence of PEG 4000 and PEG 3000 was realized [43]. PEG 3000 or 4000 were added to a RBCs lysis supernatant obtained after incubation with MFN1032 at a final concentration of 30 mM. No variation of hemoglobin OD value was observed in our conditions during incubation at 37°C for 1 h.

### Oligonucleotides and polymerase chain reactions

MFN1032 and MF37 strains were resuspended in 500 μL sterile ultrapure water. The suspension (2 μL) was then used for PCR amplification of DNA from bacterial colonies. PCR was carried out in a 25 μL reaction volume, in a GeneAmp PCR system 2400 (Perkin-Elmer Corporation, USA). Each reaction mixture contained DNA, 0.25 μL Taq polymerase (Q-Biogen, Illkrirch, France), 2.5 μL corresponding buffer, 2.5 μL primers (20 μM) and 2 μL deoxyribonucleoside triphosphate (2.5 mM). After initial denaturation for three minutes at 95°C, the reaction mixture was subjected to 35 cycles of 1 minute at 94°C, 1 minute at 41°C and two minutes at 72°C, followed by a final 3 minutes extension at 72°C. PCR primers, HRCR8092 (5'-CCITT(C/T)ATCGT(C/T)AT(C/T)GA(C/T)(C/T)T-3') and HRCT8986 (5'-CTGTCCCAGATIAICTGIGT-3') (where I indicates inosine), synthesized by Eurogentec (Angers, France), were designed to amplify the "*hrc *operon", a region of operon U of the *hrp *cluster of type I (*hrpU *operon), including the 3' end of *hrc*R (26%), *hrc*S (100%), and the 5' end of *hrc*T (42%) (approximately 897 bp) [23].

Aliquots (5 μL) of the PCR products were analyzed by electrophoresis in 1% agarose gels, stained with ethidium bromide and photographed under UV illumination.

### Cloning and sequencing the *hrc*RST PCR fragment

PCR products were cloned with the pMOSBlue blunt-ended cloning kit (Amersham/Biosciences). MOS cells were transformed and, after blue/white colony screening, clones were picked and plasmid DNA was isolated with the QIAprep Spin Miniprep Kit (Qiagen).

The PCR products were sequenced by Genome Express (France). The predicted sequences of MFN1032 *hrc*RST and MF37 *hrc*RST were submitted for BLAST queries http://www.ncbi.nlm.nih.gov/BLAST/.

### Construction of MFN1030, an *hrc*U operon-disrupted mutant of MFN1032 and MF1031, its revertant

The *hrc*RST-pMOS plasmid from MFN1032 was digested with *Eco*RI/*Hind*III and subsequently *hrc*RST fragment was inserted into the transferable suicide plasmid pME3087 (6,9 Kb) digested by the same enzymes [44]. This construction, pME3087-*hrc*RST (7,8 kb), was then introduced into *Escherichia coli *DH5α MCR cells by electroporation. Plasmids were isolated using the QIAprep Spin Miniprep Kit (Qiagen), checked by digestion with *Hind*III/*Eco*RI and transferred into the *Escherichia coli *conjugative strain S17.1 [45]. Colonies were selected for their resistance to tetracycline (20 μg/mL). MFN1032 (naturally ampicillin resistant) cells were conjugated with S17.1 cells carrying the pME3087-*hrc*RST plasmid and strains were selected for their resistance to tetracycline (20 μg/mL) and ampicillin (100 μg/mL) that corresponds to insertion of the whole plasmid via a single homologue recombinaison. One of the clones was selected and corresponded to an *hrpU *operon disruption mutant. This disruption mutant was called MFN1030. The reversion of the mutant MFN1030 was obtained after incubating MFN1030 cells on an LB agar plate for 72 hours. Of all the colonies obtained, 100 were subcultured in parallel on LB agar plates with or without tetracycline (20 μg/mL).

Colonies growing on LB agar plates without tetracycline but not on LB agar plates with tetracycline (20 μg/mL) reflect a second recombination event and an excision of the plasmid. One clone was selected and named MFN1031, a revertant of MFN1030 strain.

## Authors' contributions

DS carried out most experiments and analyzed most of the data. AM wrote the manuscript, participated in the design of the study and analyzed most of the data. GR participated in the molecular genetic studies, and participated in the design of the study. JG initiated and participated in the design of the study. NC helped set up general laboratory experimental conditions. MF and NO were involved in designing the study. All authors read and approved the final manuscript.

## References

[B1] Couillerot O Prigent-Combaret C Caballero-Mellado J Moenne-Loccoz Y *Pseudomonas fluorescens *and closely-related fluorescent pseudomonads as biocontrol agents of soil-borne phytopathogens Lett Appl Microbiol 2009 48 5 505 512 10.1111/j.1472-765X.2009.02566.x 19291210

[B2] Tourkya B Boubellouta T Dufour E Leriche F Fluorescence spectroscopy as a promising tool for a polyphasic approach to pseudomonad taxonomy Curr Microbiol 2009 58 1 39 46 10.1007/s00284-008-9263-0 18815829

[B3] Bodilis J Calbrix R Guerillon J Merieau A Pawlak B Orange N Barray S Phylogenetic relationships between environmental and clinical isolates of *Pseudomonas fluorescens *and related species deduced from 16S rRNA gene and OprF protein sequences Syst Appl Microbiol 2004 27 1 93 108 10.1078/0723-2020-00253 15053326

[B4] Meyer JM Geoffroy VA Baida N Gardan L Izard D Lemanceau P Achouak W Palleroni NJ Siderophore typing, a powerful tool for the identification of fluorescent and nonfluorescent pseudomonads Appl Environ Microbiol 2002 68 6 2745 2753 10.1128/AEM.68.6.2745-2753.2002 12039729 PMC123936

[B5] Burini JF Gugi B Merieau A Guespin-Michel JF Lipase and acidic phosphatase from the psychrotrophic bacterium *Pseudomonas fluorescens*: two enzymes whose synthesis is regulated by the growth temperature FEMS Microbiol Lett 1994 122 1-2 13 18 10.1111/j.1574-6968.1994.tb07136.x 7958764

[B6] Gugi B Orange N Hellio F Burini JF Guillou C Leriche F Guespin-Michel JF Effect of growth temperature on several exported enzyme activities in the psychrotrophic bacterium *Pseudomonas fluorescens* J Bacteriol 1991 173 12 3814 3820 1646789 10.1128/jb.173.12.3814-3820.1991 PMC208013

[B7] Picot L Abdelmoula SM Merieau A Leroux P Cazin L Orange N Feuilloley MG *Pseudomonas fluorescens *as a potential pathogen: adherence to nerve cells Microbes Infect 2001 3 12 985 995 10.1016/S1286-4579(01)01462-9 11580985

[B8] Feuilloley MGJ Mezghani-Abdelmoula S Picot L Lesouhaitier O Merieau A Guerillon J Orange N Involvement of *Pseudomonas *and related species in central nervous system infections Recent Adv Dev Microbiol 2003 7 55 71

[B9] Picot L Mezghani-Abdelmoula S Chevalier S Merieau A Lesouhaitier O Guerillon J Cazin L Orange N Feuilloley MG Regulation of the cytotoxic effects of *Pseudomonas fluorescens *by growth temperature Res Microbiol 2004 155 1 39 46 10.1016/j.resmic.2003.09.014 14759707

[B10] Chapalain A Rossignol G Lesouhaitier O Merieau A Gruffaz C Guerillon J Meyer JM Orange N Feuilloley MG Comparative study of 7 fluorescent pseudomonad clinical isolates Can J Microbiol 2008 54 1 19 27 10.1139/W07-110 18388968

[B11] Rossignol G Merieau A Guerillon J Veron W Lesouhaitier O Feuilloley MG Orange N Involvement of a phospholipase C in the hemolytic activity of a clinical strain of *Pseudomonas fluorescens* BMC Microbiol 2008 8 189 10.1186/1471-2180-8-189 18973676 PMC2613904

[B12] Rossignol G Sperandio D Guerillon J Duclairoir Poc C Soum-Soutera E Orange N Feuilloley MG Merieau A Phenotypic variation in the *Pseudomonas fluorescens *clinical strain MFN1032 Res Microbiol 2009 160 5 337 344 10.1016/j.resmic.2009.04.004 19409488

[B13] Barbieri JT Sun J *Pseudomonas aeruginosa *ExoS and ExoT Rev Physiol Biochem Pharmacol 2004 152 79 92 full_text 15375697 10.1007/s10254-004-0031-7

[B14] Frank DW The exoenzyme S regulon of *Pseudomonas aeruginosa* Mol Microbiol 1997 26 4 621 629 10.1046/j.1365-2958.1997.6251991.x 9427393

[B15] Vallis AJ Yahr TL Barbieri JT Frank DW Regulation of ExoS production and secretion by *Pseudomonas aeruginosa *in response to tissue culture conditions Infect Immun 1999 67 2 914 920 9916108 10.1128/iai.67.2.914-920.1999 PMC96404

[B16] Galan JE Collmer A Type III secretion machines: bacterial devices for protein delivery into host cells Science 1999 284 5418 1322 1328 10.1126/science.284.5418.1322 10334981

[B17] Huynh TV Dahlbeck D Staskawicz BJ Bacterial blight of soybean: regulation of a pathogen gene determining host cultivar specificity Science 1989 245 4924 1374 1377 10.1126/science.2781284 2781284

[B18] Huang HC He SY Bauer DW Collmer A The *Pseudomonas syringae pv*. syringae 61 hrpH product, an envelope protein required for elicitation of the hypersensitive response in plants J Bacteriol 1992 174 21 6878 6885 1400238 10.1128/jb.174.21.6878-6885.1992 PMC207366

[B19] Lee J Klusener B Tsiamis G Stevens C Neyt C Tampakaki AP Panopoulos NJ Noller J Weiler EW Cornelis GR HrpZ(Psph) from the plant pathogen *Pseudomonas syringae pv. phaseolicola *binds to lipid bilayers and forms an ion-conducting pore in vitro Proc Natl Acad Sci USA 2001 98 1 289 294 10.1073/pnas.011265298 11134504 PMC14583

[B20] Preston GM Bertrand N Rainey PB Type III secretion in plant growth-promoting *Pseudomonas fluorescens *SBW25 Mol Microbiol 2001 41 5 999 1014 10.1046/j.1365-2958.2001.02560.x 11555282

[B21] Ma Q Zhai Y Schneider JC Ramseier TM Saier MH Jr Protein secretion systems of *Pseudomonas aeruginosa *and *P fluorescens* Biochim Biophys Acta 2003 1611 1-2 223 233 10.1016/S0005-2736(03)00059-2 12659964

[B22] Rezzonico F Binder C Defago G Moenne-Loccoz Y The type III secretion system of biocontrol *Pseudomonas fluorescens *KD targets the phytopathogenic *Chromista Pythium ultimum *and promotes cucumber protection Mol Plant Microbe Interact 2005 18 9 991 1001 10.1094/MPMI-18-0991 16167769

[B23] Mazurier SLM Siblot S Mougel C Lemanceau P Distribution and diversity of type III secretion system-like genes in saprophytic and phytopathogenic fluorecent *Pseudomonas* FEMS Microbiology Ecology 2004 49 455 467 10.1016/j.femsec.2004.04.019 19712294

[B24] Toussaint B Delic-Attree I Vignais PM *Pseudomonas aeruginosa *contains an IHF-like protein that binds to the *algD *promoter Biochem Biophys Res Commun 1993 196 1 416 421 10.1006/bbrc.1993.2265 8216322

[B25] Dacheux D Goure J Chabert J Usson Y Attree I Pore-forming activity of type III system-secreted proteins leads to oncosis of *Pseudomonas aeruginosa*-infected macrophages Mol Microbiol 2001 40 1 76 85 10.1046/j.1365-2958.2001.02368.x 11298277

[B26] Broek D van den Chin AWTF Bloemberg GV Lugtenberg BJ Molecular nature of spontaneous modifications in *gacS *which cause colony phase variation in *Pseudomonas sp*. strain PCL1171 J Bacteriol 2005 187 2 593 600 10.1128/JB.187.2.593-600.2005 15629930 PMC543552

[B27] Hakansson S Schesser K Persson C Galyov EE Rosqvist R Homble F Wolf-Watz H The YopB protein of *Yersinia pseudotuberculosis *is essential for the translocation of Yop effector proteins across the target cell plasma membrane and displays a contact-dependent membrane disrupting activity Embo J 1996 15 21 5812 5823 8918459 PMC452329

[B28] Clerc P Baudry B Sansonetti PJ Plasmid-mediated contact haemolytic activity in *Shigella *species: correlation with penetration into HeLa cells Ann Inst Pasteur Microbiol 1986 137A 3 267 278 10.1016/S0769-2609(86)80033-3 3322171

[B29] Shaw RK Daniell S Ebel F Frankel G Knutton S EspA filament-mediated protein translocation into red blood cells Cell Microbiol 2001 3 4 213 222 10.1046/j.1462-5822.2001.00105.x 11298645

[B30] Crepin VF Martinez E Shaw RK Frankel G Daniell SJ Structural and functional properties of chimeric EspA-FliCi filaments of EPEC J Mol Biol 2008 378 1 243 250 10.1016/j.jmb.2008.02.042 18353364

[B31] Merieau A Gügi B Guespin-Michel JF Orange N Temperature regulation of lipase secretion by *Pseudomonas fluorescens *strain MF0 Appl Microbiol Biotechnol 1993 39 104 109

[B32] Gonzalez-Rodriguez N Santos JA Otero A Garcia-Lopez ML Cell-associated hemolytic activity in environmental strains of *Plesiomonas shigelloides *expressing cell-free, iron-influenced extracellular hemolysin J Food Prot 2007 70 4 885 890 17477257 10.4315/0362-028x-70.4.885

[B33] Paulsen IT Press CM Ravel J Kobayashi DY Myers GS Mavrodi DV DeBoy RT Seshadri R Ren Q Madupu R Complete genome sequence of the plant commensal *Pseudomonas fluorescens *Pf-5 Nat Biotechnol 2005 23 7 873 878 10.1038/nbt1110 15980861 PMC7416659

[B34] Vinatzer BA Jelenska J Greenberg JT Bioinformatics correctly identifies many type III secretion substrates in the plant pathogen *Pseudomonas syringae *and the biocontrol isolate *P. fluorescens *SBW25 Mol Plant Microbe Interact 2005 18 8 877 888 10.1094/MPMI-18-0877 16134900

[B35] Field TR Layton AN Bispham J Stevens MP Galyov EE Identification of novel genes and pathways affecting *Salmonella *type III secretion system 1 using a contact-dependent hemolysis assay J Bacteriol 2008 190 9 3393 3398 10.1128/JB.01189-07 18310344 PMC2347372

[B36] Hogardt M Roeder M Schreff AM Eberl L Heesemann J Expression of *Pseudomonas aeruginosa exoS *is controlled by quorum sensing and RpoS Microbiology 2004 150 Pt 4 843 851 10.1099/mic.0.26703-0 15073294

[B37] Bleves S Soscia C Nogueira-Orlandi P Lazdunski A Filloux A Quorum sensing negatively controls type III secretion regulon expression in *Pseudomonas aeruginosa *PAO1 J Bacteriol 2005 187 11 3898 3902 10.1128/JB.187.11.3898-3902.2005 15901720 PMC1112058

[B38] Soscia C Hachani A Bernadac A Filloux A Bleves S Cross talk between type III secretion and flagellar assembly systems in *Pseudomonas aeruginosa* J Bacteriol 2007 189 8 3124 3132 10.1128/JB.01677-06 17307856 PMC1855843

[B39] Chatterjee A Cui Y Yang H Collmer A Alfano JR Chatterjee AK GacA, the response regulator of a two-component system, acts as a master regulator in *Pseudomonas syringae pv. tomato *DC3000 by controlling regulatory RNA, transcriptional activators, and alternate sigma factors Mol Plant Microbe Interact 2003 16 12 1106 1117 10.1094/MPMI.2003.16.12.1106 14651344

[B40] Hojo H Koyanagi M Tanaka M Kajihara S Ohnishi K Kiba A Hikichi Y The *hrp *genes of *Pseudomonas cichorii *are essential for pathogenicity on eggplant but not on lettuce Microbiology 2008 154 Pt 10 2920 2928 10.1099/mic.0.2008/021097-0 18832299 PMC2885751

[B41] Filopon D Merieau A Bernot G Comet JP Leberre R Guery B Polack B Guespin-Michel J Epigenetic acquisition of inducibility of type III cytotoxicity in *P. aeruginosa* BMC Bioinformatics 2006 7 272 10.1186/1471-2105-7-272 16734902 PMC1488876

[B42] Mirleau P Delorme S Philippot L Meyer J Mazurier S Lemanceau P Fitness in soil and rhizosphere of *Pseudomonas fluorescens *C7R12 compared with a C7R12 mutant affected in pyoverdine synthesis and uptake FEMS Microbiol Ecol 2000 34 1 35 44 10.1111/j.1574-6941.2000.tb00752.x 11053734

[B43] Quijano JC Lemeshko VV Hemoglobin precipitation by polyethylene glycols leads to underestimation of membrane pore sizes Biochim Biophys Acta 2008 1778 12 2775 2780 10.1016/j.bbamem.2008.07.009 18692020

[B44] Schnider U Keel C Voisard C Defago G Haas D Tn5-directed cloning of *pqq *genes from *Pseudomonas fluorescens *CHA0: mutational inactivation of the genes results in overproduction of the antibiotic pyoluteorin Appl Environ Microbiol 1995 61 11 3856 3864 8526497 10.1128/aem.61.11.3856-3864.1995 PMC167690

[B45] Simon RPU Pehle A A broad host range mobilization system for in vitro genetic engineering: transposon mutagenesis in Gram-negative bacteriabiotechology 1983 1 784 790 10.1038/nbt1183-784

